# Bacterial communities in Thai ticks: revealing geographical and methodological gaps in surveillance—a 25-year scoping review

**DOI:** 10.1186/s41182-026-00950-6

**Published:** 2026-04-17

**Authors:** Artharee Rungrojn, Kittipong Chaisiri, Janjira Thaipadungpanit, Elizabeth M. Batty, Stuart D. Blacksell

**Affiliations:** 1 Mahidol Oxford Tropical Medicine Research Unit, Faculty of Tropical Medicine, Mahidol University, 420/6 Rajvithi Road, Bangkok, 10400 Thailand; 2 Faculty of Tropical Medicine, Mahidol University, 420/6 Rajvithi Road, Bangkok, 10400 Thailand; 3 Centre for Tropical Medicine and Global Health, Nuffield Department of Clinical Medicine, John Radcliffe Hospital, University of Oxford, Oxford, UK

**Keywords:** Tick, Tick-borne diseases, Microbiome, Bacteria, *Rickettsia*, *Coxiella*, Thailand

## Abstract

Ticks serve as key vectors for a diverse range of bacterial pathogens that affect humans and animals worldwide. In Thailand, a comprehensive understanding of tick-associated bacterial diversity remains limited. This scoping review synthesises published data on tick-borne bacteria across Thailand from 2001 to 2025, focusing on bacterial diversity, host–vector associations, geographic distribution, and molecular detection methods. Literature searches in NCBI, Embase, and Web of Science identified 402 studies (272 after duplicate removal), of which 39 met the inclusion criteria. Ticks were collected from animals, humans, and the environment across four zoogeographical regions. *Rhipicephalus*, *Haemaphysalis*, *Dermacentor*, and *Amblyomma* were the most commonly studied genera. Eighteen bacterial genera, including both pathogens and endosymbionts, were identified, with *Coxiella*-like endosymbionts, *Rickettsia*, *Anaplasma*, and *Ehrlichia* being the predominant genera. *Rhipicephalus* ticks exhibited the highest bacterial diversity, while *Rickettsia* spp. were the most frequently detected pathogens. Conventional PCR remained the principal diagnostic method, with limited application of quantitative and metagenomic sequencing approaches. Geographic analysis revealed that most studies were concentrated in the Northern Peninsular and Central Peninsular regions, while the Continental section of the Indo-Chinese Mainland and Korat Plateau zones were under-represented, which may limit the accuracy of regional risk assessments, as surveillance gaps can underestimate both the diversity and prevalence of pathogenic organisms in these areas. This review emphasises the intricate nature of tick–host–pathogen interactions and highlights the importance of implementing standardised genomic surveillance nationwide within a One Health framework. The findings reveal key gaps in current surveillance efforts and advocate for incorporating genomic tick monitoring into Thailand’s national One Health strategies to improve zoonotic disease preparedness.

**Supplementary Information:**

The online version contains supplementary material available at 10.1186/s41182-026-00950-6.

## Introduction

Ticks are obligate haematophagous ectoparasites that act as important vectors of pathogens affecting both animals and humans. Tick-borne diseases are recognised as a neglected public health concern in Thailand, despite their potential impact on both the veterinary and human health sectors [1]. Ticks are classified into two major families: Ixodidae (hard ticks) and Argasidae (soft ticks) [2]. Hard ticks are the predominant vectors in Thailand and play a key role in pathogen transmission [3]. They parasitise a wide range of hosts, including rodents [4], small mammals [5], domestic animals, companion animals [6], primates [7], and ectothermic species [8]. They are occasionally found as free-living individuals in the environment [9]. Ticks can transmit a variety of pathogens that cause diseases worldwide, such as Lyme disease, Rocky Mountain spotted fever, rickettsioses, anaplasmosis, and ehrlichiosis [10]. In addition to pathogenic microorganisms, ticks harbour non-pathogenic, vertically transmitted intracellular bacteria known as endosymbionts [11]. These endosymbionts are crucial for tick physiology, as they contribute to development, reproduction, and overall fitness. Notably, *Coxiella*-like endosymbionts (CLEs) and *Francisella*-like endosymbionts (FLEs) are strongly associated with tick survival and are involved in the synthesis of vitamins required for growth and reproduction [11]. Microbial detection in ticks employs a range of methodologies, including serological assays and molecular techniques, such as polymerase chain reaction (PCR), real-time PCR, and DNA sequencing. With the advancement of next-generation sequencing (NGS) technologies, tick microbiome studies have become increasingly comprehensive, enabling improved detection of both pathogenic and symbiotic microorganisms [12].

Thailand occupies a unique position within Southeast Asia, characterised by a tropical climate, a rich biodiversity, and rapid environmental transformations, including land-use modification and climate change, which influence vector biology and the incidence of vector-borne diseases [13]. Biogeographically, the country spans the transition between the Indo-Chinese and Sundaic subregions, delineated by the Isthmus of Kra. This transition zone serves as an important criterion for terrestrial mammal distribution, resulting in four distinct zoogeographical regions [14]. Understanding the environmental variables and distinct biological assemblages within these zones is essential for characterising the distribution of tick vectors and the pathogens they harbour. Molecular surveillance in Thailand has historically relied on target pathogen detection with PCR and Sanger sequencing, which are effective for detecting known, targeted pathogens but are limited in characterising entire microbial communities. Over the last two decades, the transition towards NGS and metagenomics has revolutionised the field by enabling an "unbiased" approach to pathogen discovery [15]. These high-throughput technologies facilitate the simultaneous identification of diverse bacteria, including rare pathogens and non-pathogenic endosymbionts (for instance, Intirach et al. (2024) documented the first report in Zhonghe, Danzhou, China, of *Anaplasma* sp., *A. marginale*, *Rickettsia felis*, and *Coxiella* detected in *Rhipicephalus haemaphysaloides*), providing a more comprehensive understanding of the tick microbiome than conventional, target-specific assays.

In Thailand, the risk of tick-borne zoonoses is potentially intensified at the human–animal–environment interface, particularly as forest encroachment for agriculture and tourism expands. Habitat fragmentation and increased human presence in ecological transitional zones (ecotones) increase the probability of "spillover" events, in which pathogens circulate from wildlife reservoirs to humans or domestic livestock [1, 16]. Adopting a One Health framework is essential for monitoring these ecological shifts and emerging and re-emerging tick- and other vector-borne diseases.

This scoping review aims to synthesise current knowledge on tick research in Thailand over the past 25 years (2001–2025). By evaluating the existing literature, this study maps the distribution of tick collection efforts across different zoogeographical regions and identifies the diversity of host species and their associated tick vectors. Furthermore, the study presents a list of pathogenic and endosymbiotic bacteria detected, along with their infection prevalence, and discusses the diagnostic methodologies employed for bacterial detection. In addition, the study highlights critical geographical and methodological gaps in current surveillance, providing a strategic foundation for nationwide, standardised genomic monitoring under a One Health framework.

## Materials and methods

### Search strategy

**Fig. 1:**
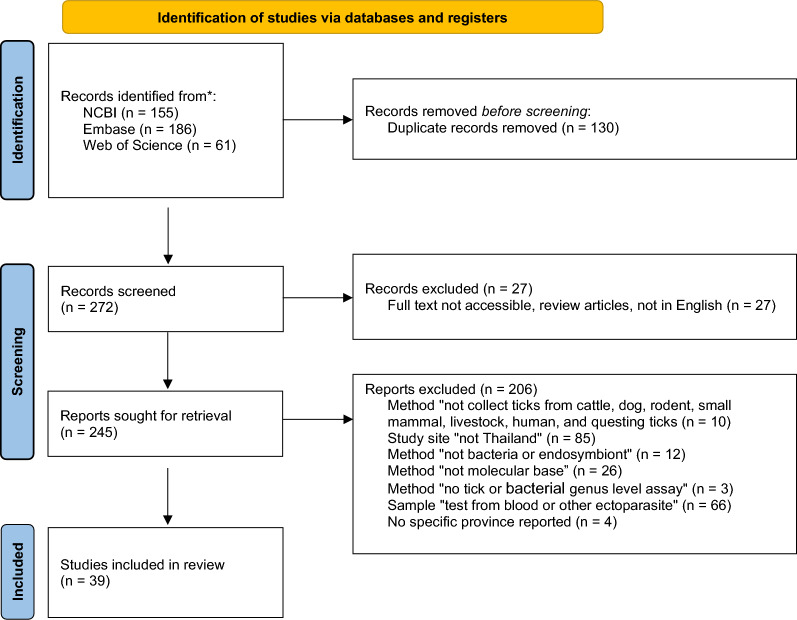
PRISMA diagram summarises the eligibility criteria used for the scoping review

A systematic search was conducted in NCBI, Embase, and Web of Science. The searches were performed between 3rd and 16th May 2025, in accordance with the PRISMA–ScR guidelines [17] (Fig. 1). The completed checklist is provided in Supplementary Table S2. We clarify that no protocol was prospectively registered in any open science framework (OSF) or repository prior to the conduct of this review. The search strings used were as follows: in NCBI PubMed, a structured search was performed using the following Boolean string applied across all fields: (‘tick’ OR ‘ticks’) AND (‘bacteria’ OR ‘pathogen’ OR ‘endosymbiont’) AND ‘Thailand’. In Embase, the search was conducted using the phrase ‘Bacteria in tick.’ In Web of Science, an advanced search was performed using the ‘All Fields search’, which searches all of the searchable fields, combining the terms ‘Bacteria,’ ‘tick,’ and ‘Thailand’ across all available fields using AND Boolean operators. Eligible studies included those that involved the collection of tick specimens from animals, humans, or questing ticks in any geographical region of Thailand [14] and provided clearly defined study sites. They reported tick species identification and/or detection of bacterial pathogens or endosymbionts. Only studies that employed molecular methods, such as sequencing-based approaches, metagenomic analyses, multiplex molecular panels, or other relevant molecular techniques, and that identified ticks and bacteria to at least the genus level were included. Furthermore, only full-text articles with accessible content were considered, with no restrictions on the year of specimen collection or publication date. Conference abstracts, theses, and preprints were excluded from the scoping review.

### Data abstraction

Inclusion and exclusion criteria.

#### Inclusion criteria

- Population: Ticks collected from animal hosts or questing within Thailand. Studies reporting *Rhipicephalus sanguineus* (sensu lato; herein referred to as *R. sanguineus*), corresponding to the tropical lineage recently reclassified as *R. linaei*, were used in this study.
- Concept: Detection of bacteria in ticks, including identification to genus and/or species level. Extracted data included author and year, province and collection site, tick species, host animal, bacterial genus/species, detection method, and the reported prevalence.
- Context: The sampling area was classified according to zoogeographical regions as described by Woodruff et al. [14]. Regions were defined using latitudinal bands and geological features. The transect was divided into four sections:
- Continental section of the Indo-Chinese Mainland (13°30′–21°N)
- Northern Peninsular (9°–13°30′N)
- Central Peninsular (6°–9°N)
- Korat Plateau (Approximately 14°-18°N)
- Study design and Methods: Original research articles reporting primary data on bacterial detection using molecular techniques and containing sufficiently detailed methodological descriptions to enable assessment of study design and data validity. Data on host categories and tick genera across zoogeographical regions were compiled by tallying the number of publications reporting each association within each region, then expressing these counts as proportions to facilitate regional comparisons. Conversely, information regarding network analyses, pathogen prevalence, and detection methods was extracted as categorical presence/absence data, indicating whether each parameter was reported in individual publications regardless of frequency or magnitude. Prevalence values were extracted directly from each study as reported by the authors. Specifically, we recorded point prevalence estimates calculated as simple proportions (number of positive ticks divided by the total number tested). Where studies reported prevalence separately by tick species, host, or location, subgroup-specific values were extracted for each subgroup. Ranges are descriptive and not directly comparable across studies due to heterogeneity in design, sampling, and diagnostics.

#### Exclusion criteria

Studies were excluded if they were conducted outside Thailand, did not involve tick samples, did not report bacterial detection in ticks, were review articles, conference abstracts without full data, editorials or opinion pieces, or lacked sufficient methodological detail or extractable data.

#### Language restrictions

No language restriction was applied during the search; however, only English-language studies were included during screening.

#### Data extraction

Data extraction was conducted by a single reviewer (Artharee Rungrojn) using a standardised extraction criteria for this study. The extracted data included publication details, sampling location, tick species, host species, detected bacterial taxa, bacterial detection methods, and reported prevalence. To ensure accuracy and consistency, extracted information and interpretations were discussed among the authors during the data synthesis process.

### Data analysis

The extracted data were compiled in Microsoft Excel LTSC MSO (Version 2408, Build 16.0.17932.20540). A map of Thailand, pie charts, bar charts, network, and statistical analysis were generated in R version 4.5.2 [18]. Animal host-tick network analysis was conducted to investigate infestation patterns using the “bipartite” package in R [19]. The network analysis was conducted to qualitatively visualise clustering patterns and shared associations among host species rather than to derive quantitative network metrics.

## Results

A total of 402 studies (2001–2025) were initially identified through systematic searches in NCBI, Embase, and Web of Science using advanced search terms related to tick-associated bacteria in Thailand. After removing duplicates, 272 unique studies were retained for screening. After screening against predefined inclusion and exclusion criteria, 39 studies were included in the final analysis (Fig. 1).

Studies were excluded for the following reasons: specimens were not collected from animals (e.g., cattle, dogs, rodents, small mammals, livestock and humans) or questing ticks (*N* = 10); study sites were located outside Thailand (*N* = 85); the study did not target bacteria or endosymbionts (*N* = 12); the methods were not molecular-based (*N* = 26); bacterial or tick identification was not performed at least to the genus level (*N* = 3); samples were tested from host blood or other ectoparasites rather than ticks (*N* = 66); or the article was inaccessible or review publications (*N* = 27).

This screening process ensured that the final set of included studies was directly relevant to the occurrence and molecular detection of bacterial pathogens in ticks across Thailand, providing a robust dataset for further analysis.

### Sample collection and publication year

**Fig. 2:**
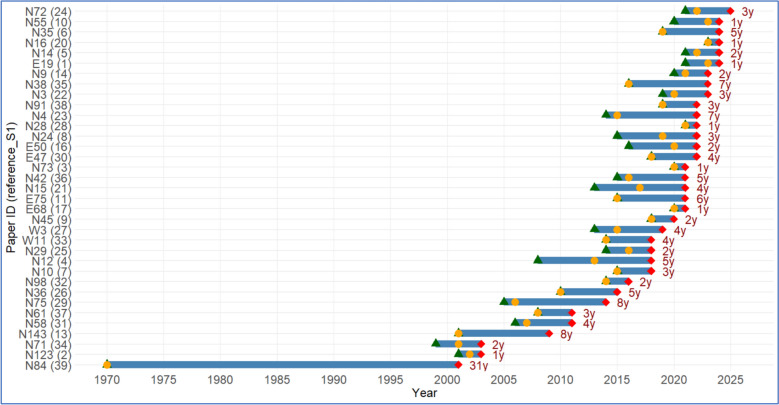
Timeline of sample collection and publication for studies of tick-borne bacteria in Thailand. Red points indicate the year of publication, and blue segments correspond to the year of sample collection

A total of 39 studies met the initial search criteria, of which 35 provided both the year of sample collection and the year of publication; the remaining four did not report the sample collection date. The time interval between sample collection and publication varied widely, with a median of 3 years (interquartile range: 2–5 years). The longest interval observed was 31 years, while the shortest was 1 year (Fig. 2). Notably, 24.3% of studies were published within 2 years of sample collection, representing the most common interval.

### Geographic distribution

**Fig. 3:**
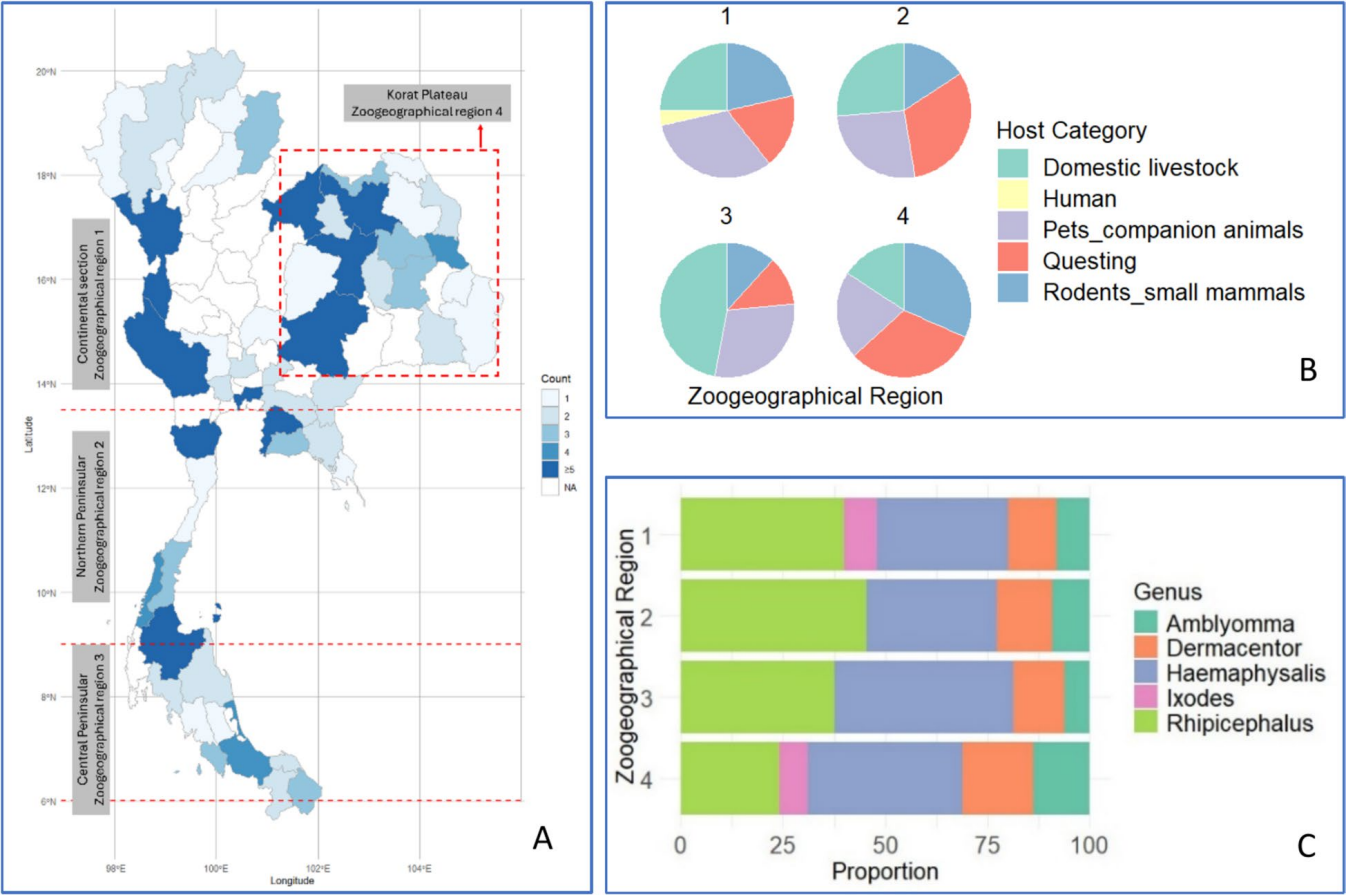
**A** Map of Thailand shows the locations of tick sample collection sites across different zoogeographical regions. It highlights areas, where sampling has taken place, ranging from regions with no previous sampling to those with over five sampling events, as indicated by the blue colour gradient in the provinces, and includes both on-host and off-host collections. **B** Proportion of the host category across zoogeographical regions. **C** Proportion of tick genus across zoogeographical regions

Based on the zoogeographical region classification of Thailand [14], the country can be divided into four distinct zoogeographical regions: 1) Continental section of the Indo-Chinese Mainland (13°30′–21°N); 2) Northern Peninsular (9°–13°30′N); 3) Central Peninsular (6°–9°N); and 4) Korat Plateau (15° 39′ 59.99" N) (Fig. 3A). Most tick-related studies have been conducted in the Northern and Central Peninsular regions (Southern Thailand), specifically in Phang Nga, Songkhla, Surat Thani, and Phetchaburi provinces. Although Nakhon Ratchasima (on the Korat Plateau) and Kanchanaburi (within the Continental section of the Indo-Chinese Mainland) were major sites for tick collection events, both the Korat Plateau and Continental section of the Indo-Chinese Mainland regions as a whole remain under-represented in tick research, as indicated by the provinces without shading in Fig. 3A.

Tick samples were obtained through two primary approaches: (i) direct collection from hosts, including small mammals (rodents), domestic livestock, companion animals, and humans, and (ii) collection of free-living (questing) ticks from the environment. In zoogeographical region 3, ticks were mainly collected directly from domestic livestock, whereas in regions 2 and 4, questing ticks and pet or companion animals accounted for equal proportions of samples. Interestingly, ticks were reported from human hosts only in region 1, based on a single study (Fig. 3B). This bar chart illustrates the proportional distribution of five tick genera across four zoogeographical regions in Thailand. The five genera represented are *Amblyomma*, *Dermacentor*, *Haemaphysalis*, *Ixodes*, and *Rhipicephalus* (Fig. 3C). *Ixodes* was documented exclusively in regions 1 and 4. In contrast, *Rhipicephalus* and *Haemaphysalis* emerged as the predominant tick genera, with widespread occurrence across all zoogeographical regions of Thailand. *Rhipicephalus* demonstrated the highest prevalence in regions 1, 2, and 3, while *Haemaphysalis* was most abundant in region 4, collectively accounting for the majority of tick genera reported throughout the country.

### Host and tick associations

**Fig. 4:**
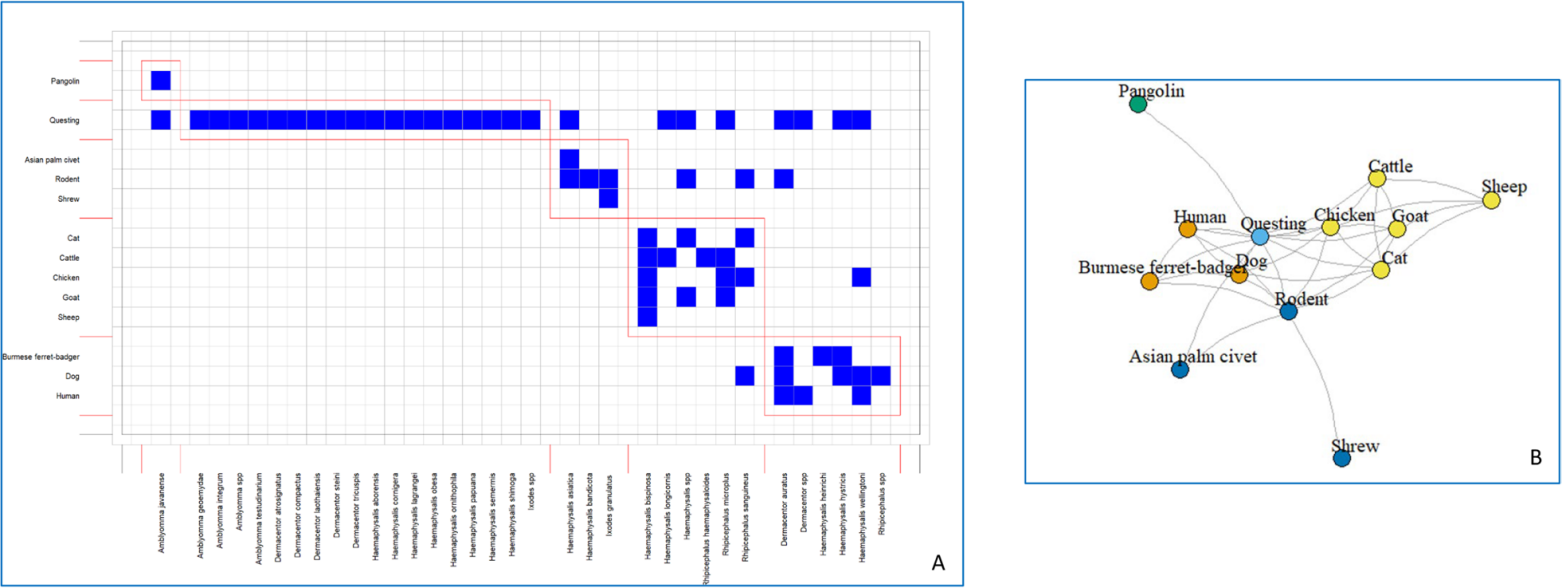
**A**, **B** Host–tick association patterns reported in Thailand. **A **Binary presence–absence matrix depicting the associations between tick species and host species. The red rectangle grouped the hosts and ticks that interact strongly. The matrix is organised with hosts categorised into five groups: (1) Burmese ferret badger, dog and human, (2) questing, (3) pangolin, (4) cat, cattle, chicken, goat, and sheep, and (5) Asian palm civet, rodent, and shrew. Blue squares indicate documented pathogen–host associations, while blank spaces represent the absence of reported associations. **B** Unipartite network illustrating relationship among host species based on shared tick species. Node colours indicate groups of hosts that are more closely interconnected through shared tick associations

A presence–absence matrix was used to visualise host–tick associations and generate a bipartite interaction network showing host species connected through shared tick species (Fig. 4A). Humans, dogs, and Burmese ferret badgers exhibited associations with particular tick species, seen as a cluster in the left portion of the matrix. *Dermacentor auratus* was identified as a connecting vector among humans, dogs, and Burmese ferret badgers, whereas *H. wellingtoni* served as a link between humans and dogs. A distinct horizontal band in the matrix indicates that certain tick species were consistently detected among questing ticks across multiple collection sites or surveys, suggesting a potential transmission risk independent of specific host identity or missing data due to publication gaps. Companion animals (cats) and domestic livestock (cattle, chickens, goats, sheep) exhibit varied association patterns, with some tick species showing specificity for livestock species. In contrast, others are shared across companion animals and with rodents. Rodents and small mammals, including the Asian palm civet and shrews, exhibited distinct tick species associations concentrated in the right portion of the matrix. *Dermacentor auratus*, previously identified as a vector linking humans, dogs, and Burmese ferret badgers, was also collected from rodents, suggesting a broader host range and a potential bridging role between sylvatic and domestic cycles. Notably, *Rhipicephalus sanguineus* and *Haemaphysalis* spp. were repeatedly found on companion animals and domestic livestock. Given their documented ability to harbour and transmit *Rickettsia* spp. and *Ehrlichia* spp., these host groups may contribute to pathogen circulation at the wildlife–livestock interface.

The unipartite network plot (Fig. 4B) depicts host species and their interconnections based on shared tick species. Nodes represent individual host species, and edges connect pairs of hosts that share at least one tick species. Node colours indicate distinct communities, representing groups of hosts that are more frequently interconnected through shared tick associations. Dogs, humans, rodents, and questing ticks are clustered at the network centre. Dogs form strong bonds with humans, wildlife (e.g., the Burmese ferret badger), and livestock. Questing ticks form a key central node, reflecting the integration of ticks collected from both hosts and the environment. In contrast, peripheral hosts such as pangolins and shrews exhibit limited connectivity.

### Bacterial genera identified from ticks in Thailand

Of the 39 documented data points, 18 genera were observed. The prevalence of bacterial genera detected in different tick genera varied widely across studies (see Table 1 and Table S1 for detailed prevalence values by tick and bacterial genus). *Rhipicephalus* ticks exhibited the highest diversity of bacterial associations, with prevalence ranging from 0.5% to 100% depending on the pathogen. Notably, *Anaplasma* (0.7–90.9%), *Bartonella* (1.3–2.5%), *Borrelia* (4.4%), *Coxiella* (1.6–50%), *Ehrlichia* (0.7–22%) were detected in *Rhipicephalus* spp. *Haemaphysalis* ticks also harboured a broad spectrum of bacteria, including *Anaplasma* (1.7–94.8%), *Borrelia* (11.5–30.5%), *Coxiella* (8.3–100%), *Ehrlichia* (0.2–1.3%), and *Rickettsia* (2–50%). *Dermacentor* ticks were primarily associated with *Anaplasma* (0.6–63.6%), *Coxiella* (2.7–33.3%), *Ehrlichia* (~ 1.4%), and *Rickettsia* (1.7–33.3%). *Amblyomma* ticks were most commonly infected with *Anaplasma* (2.3–29.6%), *Coxiella* (3.3–100%), *Ehrlichia* (8.2%), and *Rickettsia* (18.2–100%). The available data indicate a narrower prevalence range of *Rickettsia* (2.4–16.7%) in *Ixodes*.

**Table 1:** Summary of bacterial diversity, host range, and prevalence detected in major tick genera in Thailand (2001–2025)

Tick genus	No. of studies	Host types sampled	No. of bacterial genera detected	Dominant bacterial genera	Reported prevalence range (%) of dominant bacterial genera	Common detection methods
*Amblyomma*	Moderate	Pangolin, questing	4	*Anaplasma*, *Coxiella*, *Ehrlichia*, *Rickettsia*	2.3–29.6, 3.3–100, 8.2^P^, 18.2–100	PCR, Sanger sequencing, NGS (Illumina)
*Dermacentor*	Moderate	Dog, questing	4	*Anaplasma*, *Coxiella*, *Ehrlichia*, *Rickettsia*	0.6–63.6, 2.7–33.3, 1.4^P^, 1.7–33.3	PCR, Sanger sequencing, NGS (Illumina)
*Haemaphysalis*	High	Asian palm civet, Burmese ferret badger, Chicken, Dog, Goat, Rodent, questing	8	*Anaplasma*, *Bartonella*, *Borrelia*, *Coxiella*, *Ehrlichia*, *Rickettsia*	1.7^P^-94.8, 0.08^MLE^, 11.5^P^-30.5^P^, 8.3–100, 0.2–1.3^P^, 2P-50^P^	PCR, Sanger sequencing, NGS (Illumina)
*Ixodes*	Low	Rodent	1	*Rickettsia*	2.4–16.7^P^	PCR, Sanger sequencing
*Rhipicephalus*	High	Cattle, Chicken, Dog, Rodent, questing	9	*Anaplasma*, *Bartonella*, *Borrelia*, *Coxiella*, *Ehrlichia*	0.7–90.9, 1.3–2.5, 4.4^P^, 1.6–50, 0.7^P^-22	RFLP, qPCR, PCR, Sanger sequencing, NGS (Illumina)

The prevalence shown in the table represents single infections, indicating the proportion of individual ticks infected with bacterial genera within each tick genus. ^P^ indicates pooled prevalence, ^MLE^ maximum likelihood estimator

### Detection methods

**Fig. 5:**
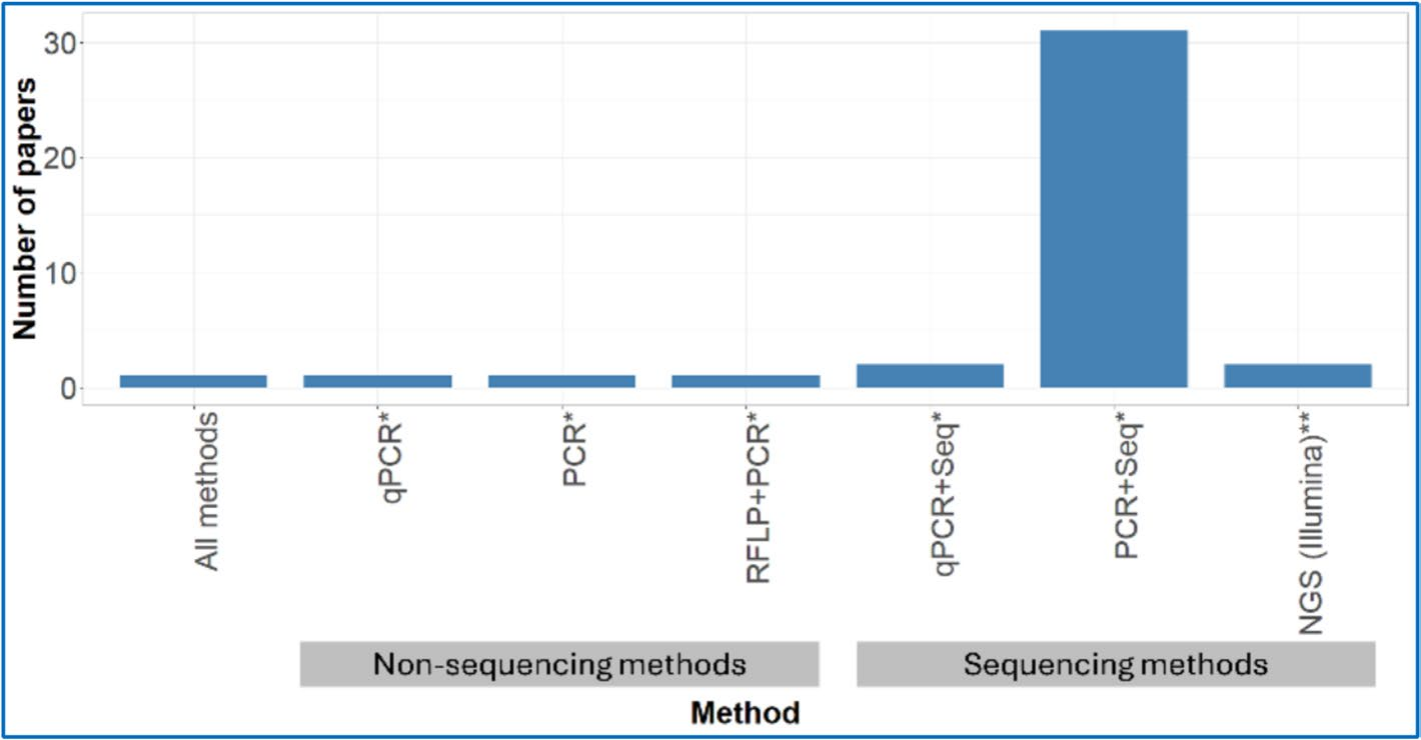
Comparative distribution of publications across different PCR-based methodologies, revealing a striking predominance of one approach in the scientific literature. * Indicates *PCR-based targeted assays (e.g., genus-specific PCR)*, whereas ** denotes non-targeted metagenomic approaches (NGS)

There are five main bacterial detection methods reported in the 39 studies, with counts per method recorded per study: restriction fragment length polymorphisms (RFLP), quantitative real-time PCR (qPCR), conventional or nested PCR (PCR), Sanger sequencing (Seq), and next-generation sequencing (NGS-Illumina). The results demonstrate that PCR combined with Sanger sequencing (PCR + Seq) accounts for the vast majority of publications in this analysis, with approximately 31 papers, representing roughly 79% of the total dataset. In contrast, the remaining methodologies show considerably lower publication frequencies, each contributing between 1 and 3 papers. PCR-based approaches became the most commonly used detection methods in studies published after the early 2000s. In more recent years, NGS has also been increasingly reported among the included studies (Fig. 5).

Different genetic markers were employed to detect the target bacterial genera. The *gro*EL genes (60 kDa heat shock protein) [9] and the internal transcribed spacer (*ITS*) region [20] were used for the detection of *Coxiella* spp. and *Bartonella* spp., respectively. Detection of *Rickettsia* spp. was performed using fragments of the 17 kDa antigen gene, *gltA*, and *ompA* genes [9]. *Anaplasma* spp., *Borrelia* spp., *Ehrlichia* spp., and *Wolbachia* spp. were identified using the 16S rRNA gene fragment [21, 22].

## Discussion

From an initial pool of 402 publications retrieved from NCBI, Embase, and Web of Science, we identified 39 studies relevant to bacteria associated with ticks across Thailand. Our analysis focused on six key aspects: (1) sampling collection and year of publication. (2) the geographical distribution of tick collection sites across different zoogeographical regions; (3) the diversity of host species associated with tick vectors; (4) the identification of pathogenic and endosymbiotic bacteria detected in ticks; (5) the methodologies employed for bacterial detection; and (6) the reported infection and prevalence rates across studies.

Although no specific guidelines exist for the allowable interval between sample collection and publication, the median data age at publication for clinical trials in high-impact journals has been reported to be approximately 3 years [23]. We draw this comparison as a contextual analogy to highlight publication timelines rather than as a formal benchmark applicable to tick-borne bacterial studies. This finding is consistent with our observations on tick-borne bacterial studies, where the median data age was also 3 years. However, the time span across studies in our dataset varied considerably, ranging from 1 to 31 years. The timing of sample collection is critical, as the interval between collection and processing directly impacts sample quality and the reliability of downstream analytical results. The publication year is crucial for determining the relevance and current validity of the findings [24, 25], as older findings may no longer be applicable due to changes in the ecology of ticks and host species over time, and due to scientific advancement rendering older molecular techniques outdated. Moreover, existing evidence is largely limited to cross-sectional studies, which provide only snapshots of pathogen presence and prevalence. The absence of long-term studies restricts understanding of temporal dynamics among hosts, vectors, ticks, and bacteria, as well as trends in distribution and infection linked to ecological or environmental factors.

The distribution pattern of tick collections across Thailand reflects both the country’s ecological diversity and variations in sampling accessibility among different zoogeographical regions. The Central and Northern peninsular regions (zones 2 and 3), encompassing much of southern Thailand on the Malay Peninsula, exhibited the highest frequency of tick collections. This pattern may be attributed to the discontinuities and disjunct series of mountains, the complex biogeographical nature of fauna, and favourable microclimatic conditions that promote tick survival and host abundance in these areas [26]. Moreover, bias arising from the selection of sampling areas may also be present. In contrast, the Korat Plateau showed a moderate level of tick collection, likely influenced by its mixed land-use patterns and variable host availability [27]. The Continental section of the Indo-Chinese Mainland, which covers most of Thailand’s northern and central regions, recorded the fewest collections. This limited sampling effort may reflect differences in host population density, economic activities, and environmental conditions far from the border area [28]. Moreover, in the report of Arnuphapprasert et al. (2023), the prevalence of Anaplasmosis in cattle in northern and central Thailand (zoogeographical region 1) ranges from 19.1% to 39.3%, representing the lowest rate compared with other regions of the country [29]. This may partially explain the comparatively limited research focus and sampling effort in this region relative to other areas of Thailand, particularly where certain pathogens (e.g., *A. marginale*) have been reported at lower prevalence. However, lower reported prevalence of a specific pathogen does not necessarily indicate reduced overall tick-borne bacterial risk. Importantly, undersampling itself represents a major limitation for inferring regional risk, as limited surveillance may underestimate pathogen diversity and prevalence. Consequently, apparent differences between regions may reflect variation in sampling intensity, study design, and diagnostic approaches rather than true epidemiological patterns.

The host–tick associations identified across Thailand highlight complex ecological interactions shaped by host availability, habitat diversity, and vector adaptability. The predominance of *R. microplus*, *H. longicornis,* and *R. sanguineus* in samples collected from cattle and dogs aligns with their known host preferences and widespread occurrence in tropical and subtropical regions (Fig. 4). These species are recognised vectors of significant veterinary and zoonotic pathogens, underscoring their epidemiological importance [30, 31]. The detection of seven overlapping tick species—*A. javanense*, *D. auratus*, *H. asiatica*, *H. hystricis*, *H. longicornis*, *H. wellingtoni*, and *R. microplus*—in both questing and host-attached collections suggests broad ecological plasticity and host-seeking behaviour. These taxa are likely to facilitate pathogen spillover among wildlife, livestock, and humans, particularly where agricultural and forest ecosystems converge [1, 3, 32, 33]. The presence of multiple tick species on hosts such as dogs, cats, and the Burmese ferret badger indicates broader exposure to diverse tick populations and habitats. Conversely, the Asian palm civet, pangolin, sheep, and shrew each harbour a single tick species. Such restricted associations may reflect host-ectoparasite coevolution or sampling limitations in specific habitats. The dominance of *R. microplus* in cattle [34], *R. sanguineus* in dogs [35, 36], and *H. wellingtoni* in poultry [37] is consistent with previous regional findings, reinforcing their status as primary ectoparasites in domestic environments. Rodents, primarily infested with *H. bandicota* and *I. granulatus*, represent potential reservoirs for zoonotic agents, including *Rickettsia* and *Bartonella* spp., as well as *Borrelia* spp. [4, 22, 38–40].

In the unipartite network plot, the central group comprising dogs, humans, rodents, and questing ticks shows extensive sharing of tick species, supporting their potential roles as bridging hosts or sentinels in tick-borne pathogen transmission. Dogs, in particular, may represent a key interface for pathogen spillover among domestic animals, wildlife, and humans, consistent with their close contact with multiple host groups [41–43]. The central position of questing ticks highlights the contribution of environmental tick populations in linking host species within the transmission network [44]. In contrast, hosts with lower connectivity appear to have more host-specific tick associations, suggesting distinct ecological roles that merit further investigation or sampling limitations in some animal groups, particularly wildlife.

This study reveals that *Haemaphysalis* and *Rhipicephalus* ticks serve as major bacterial carriers, with consistently high prevalences across multiple genera, underscoring their significant epidemiological importance in tick-borne bacterial transmission cycles. *Rickettsia* spp. were the most frequently detected pathogenic bacteria, consistent with reports from other Southeast Asian countries [45]. The predominance of *Rickettsia* may be attributed to their high prevalence in *Amblyomma* ticks, as well as the availability of sensitive and specific PCR assays for their detection [32]. The detection of pathogenic *Rickettsia* species in these vectors raises public health concerns, as several *Rickettsia* spp. are known causative agents of rickettsioses, which can cause severe illness in humans and animals. The presence of *Anaplasma*, *Coxiella*, and *Ehrlichia* in our samples highlights the risk of anaplasmosis, Q fever, and ehrlichiosis transmission, diseases that affect both livestock productivity and human health in endemic regions [1]. *Coxiella*-like and *Francisella*-like endosymbionts were also commonly identified, albeit in non-pathogenic contexts [46, 47]. In addition, *Wolbachia* is widely distributed in nature and infects terrestrial arthropod species [48]. While these endosymbionts are not directly pathogenic, emerging evidence suggests they may play crucial roles in tick biology by influencing nutrient provisioning, immune function, and potentially modulating vector competence for pathogenic bacteria [11]. Their potential roles in influencing pathogen acquisition and tick biology remain insufficiently investigated in Thai tick populations.

Microbial detection in ticks employs a variety of methodologies, including serological assays and molecular techniques, such as PCR, real-time PCR, and DNA sequencing [49]. The advent of NGS and third-generation sequencing technologies has significantly enhanced the scope and precision of tick metagenomic studies, allowing for more comprehensive detection of both pathogenic and symbiotic microorganisms [50, 51]. As demonstrated in *Rhipicephalus* spp. ticks (Fig. 4), NGS-based approaches reveal a greater bacterial diversity compared to conventional PCR methods [52]. Here, the evolution of microbial detection in Thai ticks reveals a significant reliance on traditional molecular techniques, potentially biasing our current understanding of tick-associated bacteria. While the global trend has shifted towards high-throughput platforms, our analysis shows that conventional or PCR + Seq remains the dominant methodology, accounting for 79% (31/39) of the included studies. However, this approach is robust for identifying specific, high-load pathogens, such as *Rickettsia* spp. via *gltA* or *ompA* markers, and it inherently lacks the resolution to characterise the hidden communities of the tick microbiome. The limited application of NGS, reflected in only a few studies in this review, suggests that the true bacterial diversity in Thai ticks remains under-reported. As seen in the higher diversity indices reported in *Rhipicephalus spp*., when using metagenomic approaches, non-targeted detection using NGS helps bypass the limitations of bias associated with targeted taxa-specific markers. To move towards a modern "One Health" surveillance framework, it is necessary to transition from targeted-pathogen assays to unbiased metagenomic and amplicon sequencing approaches. This shift will be crucial for uncovering complex multi-microbial interactions and identifying emerging zoonotic agents and arthropod symbionts that conventional PCR might overlook.

In alignment with the Quadripartite Organisations (FAO, UNEP, WOAH, and WHO) One Health Joint Plan of Action (2022–2026), our findings highlight the need for integrated tick surveillance encompassing human, wildlife, domestic animal, vector, and environmental health in Thailand [53]. In practice, genomic monitoring of tick-associated bacteria could be incorporated into existing zoonotic disease surveillance coordinated by the Department of Disease Control, Thailand and the Department of Livestock Development, Thailand, which jointly oversee surveillance and control of zoonotic diseases affecting human and animal populations in Thailand [54]. Tick sampling from livestock, companion animals and wildlife interfaces could be linked with molecular screening of febrile illness cohorts in hospitals to better identify potential zoonotic transmission pathways. Effective implementation would also require structured data management systems and intersectoral data sharing platforms to integrate genomic, epidemiological, and ecological data across human, animal and environmental health sectors. Such coordinated data governance would facilitate timely risk assessment, improve outbreak detection, and support evidence-based decision-making. However, several barriers still remain and potentially limit the widespread implementation of next-generation sequencing, including costs, specialised laboratory infrastructure, and bioinformatic capacity. As sequencing costs decline and national genomic capacity expands, integrating metagenomic or amplicon-based sequencing approaches could substantially enhance the detection of emerging pathogens and tick-associated symbionts within a One Health surveillance framework.

This study has several limitations. This scoping review was conducted without prospective protocol registration in a recognised registry (e.g., PROSPERO or OSF). This review was restricted to studies published in English, which may have introduced language bias and resulted in the exclusion of relevant research published in Thai or other regional languages. As a consequence, some locally conducted studies, reports in national journals, or surveillance findings may not have been captured, potentially leading to an under-representation of tick research conducted in Thailand. Language restrictions can, therefore, influence the perceived geographic distribution of studies and the diversity of reported pathogens, particularly in regions, where locally published research may not be indexed in international databases. The literature search was restricted to three databases, NCBI, Embase, and Web of Science, potentially omitting relevant studies. Moreover, the search strategy focused specifically on keywords related to bacteria in ticks from Thailand, potentially excluding studies that used broader or alternative terminology. Publications employing non-molecular detection methods were also excluded. In addition, many of the underlying studies relied on targeted PCR assays that detect only specific bacterial genera, potentially underestimating overall bacterial diversity and prevalence. Sampling in several studies was limited, both in terms of host range and sample size, reducing the generalizability of the findings. Finally, only a single confirmed record of this tick species was collected directly from a human host, underscoring the need to document the extent and nature of human-vector contact.

## Conclusion

Ticks in Thailand harbour diverse bacterial microbiomes predominantly composed of *Rickettsia* and *Coxiella*-like endosymbionts. Although PCR-based detection methods have yielded valuable insights into tick-associated bacterial communities, targeted PCR approaches may fail to detect numerous bacterial genera, thereby limiting comprehensive genomic surveillance. Long-read NGS provides sufficient sequence coverage and read length to enable reliable species-level identification of tick-borne bacteria, thereby improving the standard of genomic surveillance for the field of bacterial community studies in ticks in Thailand. The implementation of genetic surveillance to characterise bacterial communities across animal, environmental, and human interfaces within a One Health framework will enhance our understanding of disease aetiology and inform the development of improved prevention strategies for sustainable health outcomes. A standardised, multicentre NGS-based tick surveillance platform in Thailand could serve as a regional model for Southeast Asia.

## Supplementary Information

Additional file 1.

Additional file 2.

## Data Availability

No datasets were generated or analysed during the current study.
