# Supplementary material for: Bacterial communities in Thai ticks: revealing geographical and methodological gaps in surveillance—a 25-year scoping review
Source: Trop Med Health. 2026 Apr 17;54:72. doi: 10.1186/s41182-026-00950-6 (PMC13088644; doi:10.1186/s41182-026-00950-6)
Supplement: Supplementary file 1 — Additional file 1. [file 41182_2026_950_MOESM1_ESM.docx]

**Supplementary table S1:** The prevalence shown in the table represents single infections, indicating the proportion of individual ticks infected with bacterial genera within each tick genus. “NA” indicates data not available, MLE is the Maximum Likelihood Estimator.

| **Bacteria genus** | **Tick genus** | **Pool/individual** | **Prevalence (%)** | **MLE (%)** | **Province** | **Zoogeographical region** | **Paper ID** | **Reference** |
| --- | --- | --- | --- | --- | --- | --- | --- | --- |
| *Anaplasma* | *Amblyomma* | pool | NA | 1.06 | Chon Buri | 2 | E19 | (1) |
| *Anaplasma* | *Amblyomma* | individual | 29.6 | NA | Kanchanaburi | 1 | N123 | (2) |
| *Anaplasma* | *Amblyomma* | individual | 2.3 | NA | Nakhon Ratchasima | 4 | N73 | (3) |
| *Anaplasma* | *Dermacentor* | individual | 8.1 | NA | Nakhon Nayok | 1 | N12 | (4) |
| *Anaplasma* | *Dermacentor* | individual | 0.6 | NA | Chanthaburi | 2 | N12 | (4) |
| *Anaplasma* | *Dermacentor* | individual | 60 | NA | Phetchaburi | 2 | N12 | (4) |
| *Anaplasma* | *Dermacentor* | individual | 63.6 | NA | Tak | 1 | N12 | (4) |
| *Anaplasma* | *Dermacentor* | individual | 22.2 | NA | Kanchanaburi | 1 | N12 | (4) |
| *Anaplasma* | *Dermacentor* | individual | 45.5 | NA | Sa Kaeo | 4 | N12 | (4) |
| *Anaplasma* | *Dermacentor* | individual | 50 | NA | Prachuap Khiri Khan | 2 | N12 | (4) |
| *Anaplasma* | *Dermacentor* | individual | 15 | NA | Kanchanaburi | 1 | N123 | (2) |
| *Anaplasma* | *Dermacentor* | individual | 33.3 | NA | Chon Buri | 2 | N14 | (5) |
| *Anaplasma* | *Dermacentor* | pool | 1.4 | NA | Nakhon Ratchasima | 4 | N35 | (6) |
| *Anaplasma* | *Haemaphysalis* | individual | 1.9 | NA | Chon Buri | 2 | N10 | (7) |
| *Anaplasma* | *Haemaphysalis* | pool | 2.9 | NA | Chon Buri | 2 | N10 | (7) |
| *Anaplasma* | *Haemaphysalis* | individual | 94.8 | NA | Chon Buri | 2 | N14 | (5) |
| *Anaplasma* | *Haemaphysalis* | individual | 14.3 | NA | Loei | 4 | N24 | (8) |
| *Anaplasma* | *Haemaphysalis* | pool | 1.7 | NA | Nakhon Ratchasima | 4 | N35 | (6) |
| *Anaplasma* | *Haemaphysalis* | individual | 8.3 | NA | Songkhla | 3 | N73 | (3) |
| *Anaplasma* | *Haemaphysalis* | individual | 1.8 | NA | Nakhon Ratchasima | 4 | N73 | (3) |
| *Anaplasma* | *Haemaphysalis* | pool | 0 | NA | Nakhon Ratchasima | 4 | N45 | (9) |
| *Anaplasma* | *Haemaphysalis* | individual | 8.3 | NA | Songkhla | 3 | N55 | (10) |
| *Anaplasma* | *Haemaphysalis* | individual | 8.3 | NA | Songkhla | 3 | N55 | (10) |
| *Anaplasma* | *Rhipicephalus* | individual | 0.7 | NA | Chon Buri | 2 | N10 | (7) |
| *Anaplasma* | *Rhipicephalus* | individual | 8.2 | NA | Bangkok | 1 | E75 | (11) |
| *Anaplasma* | *Rhipicephalus* | pool | 1.5 | NA | Narathiwat | 4 | E113 | (12) |
| *Anaplasma* | *Rhipicephalus* | pool | 2.1 | NA | Phayao | 1 | E113 | (12) |
| *Anaplasma* | *Rhipicephalus* | individual | 90.9 | NA | Chon Buri | 2 | N14 | (5) |
| *Anaplasma* | *Rhipicephalus* | individual | 2.3 | NA | Bangkok | 1 | N143 | (13) |
| *Anaplasma* | *Rhipicephalus* | individual | 6.25 | NA | Khon Kaen | 4 | N9 | (14) |
| *Anaplasma* | *Rhipicephalus* | individual | 12.5 | NA | Sakon Nakhon | 4 | N9 | (14) |
| *Anaplasma* | *Rhipicephalus* | individual | 7.14 | NA | Kalasin | 4 | N9 | (14) |
| *Anaplasma* | *Rhipicephalus* | individual | 28.57 | NA | Mukdahan | 4 | N9 | (14) |
| *Anaplasma* | *Rhipicephalus* | individual | 27.78 | NA | Nakhon Phanom | 4 | N9 | (14) |
| *Anaplasma* | *Rhipicephalus* | individual | 39.13 | NA | Loei | 4 | N9 | (14) |
| *Anaplasma* | *Rhipicephalus* | individual | 27.27 | NA | Bueng Kan | 4 | N9 | (14) |
| *Anaplasma* | *Rhipicephalus* | individual | 28.57 | NA | Udon Thani | 4 | N9 | (14) |
| *Anaplasma* | *Rhipicephalus* | individual | 16.67 | NA | Maha Sarakham | 4 | N9 | (14) |
| *Anaplasma* | *Rhipicephalus* | individual | 25 | NA | Nong Bua Lam Phu | 4 | N9 | (14) |
| *Anaplasma* | *Rhipicephalus* | individual | 50 | NA | Nong Khai | 4 | N9 | (14) |
| *Anaplasma* | *Rhipicephalus* | individual | 12.5 | NA | Khon Kaen | 4 | N9 | (14) |
| *Anaplasma* | *Rhipicephalus* | individual | 9.5 | NA | Roi Et | 4 | N9 | (14) |
| *Anaplasma* | *Rhipicephalus* | individual | 21.43 | NA | Mukdahan | 4 | N9 | (14) |
| *Anaplasma* | *Rhipicephalus* | individual | 4.35 | NA | Loei | 4 | N9 | (14) |
| *Anaplasma* | *Rhipicephalus* | individual | 14.29 | NA | Udon Thani | 4 | N9 | (14) |
| *Anaplasma* | *Rhipicephalus* | individual | 20 | NA | Nong Khai | 4 | N9 | (14) |
| *Anaplasma* | *Rhipicephalus* | individual | 0 | NA | Phetchaburi | 2 | N55 | (10) |
| *Anaplasma* | *Rhipicephalus* | individual | NA | NA | Mukdahan, Udon Thani,  Kalasin | 4 | N65 | (15) |
| *Anaplasma* | *Rhipicephalus* | pool | 0 | NA | Phetchaburi | 2 | E50 | (16) |
| *Anaplasma* | *Rhipicephalus* | Individual | 0 | NA | Maha Sarakham, Amnat Chareon, Nakhon Ratchasima, Bangkok | 4,1 | E68 | (17) |
| *Bartonella* | *Haemaphysalis* | pool | NA | 0.08 | Chon Buri | 2 | E19 | (1) |
| *Bartonella* | *Haemaphysalis* | pool | 0 | NA | Nakhon Ratchasima | 4 | N45 | (9) |
| *Bartonella* | *Rhipicephalus* | individual | 2.5 | NA | Bangkok | 1 | N32 | (18) |
| *Bartonella* | *Rhipicephalus* | individual | 1.3 | NA | Khon Kaen | 4 | N89 | (19) |
| *Bartonella* | *Rhipicephalus* | pool | 0 | NA | Chachoengsao | 1 | N16 | (20) |
| *Borrelia* | *Haemaphysalis* | pool | 30.5 | NA | Rayong | 2 | N15 | (21) |
| *Borrelia* | *Haemaphysalis* | pool | 11.5 | 23.8 | Phayao | 1 | N15 | (21) |
| *Borrelia* | *Haemaphysalis+Ixodes* | pool | 22.2 | 14.53 | Tak | 1 | N3 | (22) |
| *Borrelia* | *Rhipicephalus* | pool | 4.4 | NA | Rayong | 2 | N15 | (21) |
| *Castellaniella* | *Rhipicephalus* | individual | NA | NA | Nakhon Phanom | 4 | N65 | (15) |
| *Castellaniella* | *Haemaphysalis* | individual | NA | NA | Udon Thani | 4 | N65 | (15) |
| *Corynebacterium* | *Rhipicephalus* | individual | NA | NA | Udon Thani, Kalasin, Loei | 4 | N65 | (15) |
| *Corynebacterium* | *Haemaphysalis* | individual | NA | NA | Nakhon Phanom, Udon Thani | 4 | N65 | (15) |
| *Coxiella* | *Amblyomma* | individual | 3.3 | NA | Nakhon Nayok | 1 | N12 | (4) |
| *Coxiella* | *Amblyomma* | individual | 50 | NA | Chanthaburi | 2 | N12 | (4) |
| *Coxiella* | *Amblyomma* | individual | 100 | NA | Chaiyaphum | 4 | N4 | (23) |
| *Coxiella* | *Amblyomma* | individual | 75 | NA | Songkhla | 3 | N72 | (24) |
| *Coxiella* | *Dermacentor* | individual | 2.7 | NA | Nakhon Nayok | 1 | N12 | (4) |
| *Coxiella* | *Dermacentor* | individual | 22.2 | NA | Kanchanaburi | 1 | N12 | (4) |
| *Coxiella* | *Dermacentor* | individual | 33.3 | NA | Chiang Mai | 1 | N12 | (4) |
| *Coxiella* | *Dermacentor* | individual | 9.8 | NA | Songkhla | 3 | N72 | (24) |
| *Coxiella* | *Haemaphysalis* | individual | 40 | NA | Chiang Rai | 1 | N24 | (8) |
| *Coxiella* | *Haemaphysalis* | individual | 14.3 | NA | Loei | 4 | N24 | (8) |
| *Coxiella* | *Haemaphysalis* | individual | 33.3 | NA | Chumphon | 2 | N29 | (25) |
| *Coxiella* | *Haemaphysalis* | individual | 37.5 | NA | Krabi | 3 | N29 | (25) |
| *Coxiella* | *Haemaphysalis* | individual | 100 | NA | Rayong | 2 | N29 | (25) |
| *Coxiella* | *Haemaphysalis* | individual | 8.3 | NA | Satun | 3 | N29 | (25) |
| *Coxiella* | *Haemaphysalis* | individual | 100 | NA | Trang | 3 | N29 | (25) |
| *Coxiella* | *Haemaphysalis* | individual | 30 | NA | Nakhon Ratchasima | 4 | N15 | (26) |
| *Coxiella* | *Haemaphysalis* | individual | 46.2 | NA | Phetchaburi | 2 | N36 | (26) |
| *Coxiella* | *Haemaphysalis* | individual | 93.3 | NA | Chanthaburi | 2 | N36 | (26) |
| *Coxiella* | *Haemaphysalis* | individual | 26.9 | NA | Tak | 1 | N36 | (26) |
| *Coxiella* | *Haemaphysalis* | individual | 53.9 | NA | Sa Kaeo | 2 | N36 | (26) |
| *Coxiella* | *Haemaphysalis* | individual | 100 | NA | Chaiyaphum | 4 | N4 | (23) |
| *Coxiella* | *Haemaphysalis* | individual | 59.5 | NA | Songkhla | 3 | N72 | (24) |
| *Coxiella* | *Haemaphysalis* | individual | 64.3 | NA | Pattani | 3 | W3 | (27) |
| *Coxiella* | *Haemaphysalis* | individual | 75 | NA | Phangnga | 3 | W3 | (27) |
| *Coxiella* | *Haemaphysalis* | individual | 100 | NA | Satun | 3 | W3 | (27) |
| *Coxiella* | *Haemaphysalis* | individual | 100 | NA | Songkhla | 3 | W3 | (27) |
| *Coxiella* | *Haemaphysalis* | individual | 100 | NA | Yala | 3 | W3 | (27) |
| *Coxiella* | *Haemaphysalis* | individual | 33.3 | NA | Narathiwat | 3 | W3 | (27) |
| *Coxiella* | *Haemaphysalis* | individual | NA | NA | Nakhon Phanom, Udon Thani | 4 | N65 | (15) |
| *Coxiella* | *Rhipicephalus* | individual | 50 | NA | Phra Nakhon Si Ayutthaya | 1 | N28 | (28) |
| *Coxiella* | *Rhipicephalus* | pool | 50 | NA | Phatthalung | 3 | N28 | (28) |
| *Coxiella* | *Rhipicephalus* | pool | 33.3 | NA | Ranong | 2 | N28 | (28) |
| *Coxiella* | *Rhipicephalus* | individual | 1.6 | NA | Phrae and Lamphun | 1 | N75 | (29) |
| *Coxiella* | *Rhipicephalus* | pool | 0 | NA | Chachoengsao | 1 | N16 | (20) |
| *Coxiella* | *Rhipicephalus* | individual | NA | NA | Khon Kaen, Mukdahan, Roi Et, Udon Thani, Nakhon Phanom,  Kalasin, Loei | 4 | N65 | (15) |
| *Dietzia* | *Rhipicephalus* | individual | NA | NA | Nakhon Phanom | 4 | N65 | (15) |
| *Ehrlichia* | *Amblyomma* | pool | 8.2 | NA | Nakhon Ratchasima | 4 | N35 | (6) |
| *Ehrlichia* | *Dermacentor* | pool | 1.4 | NA | Nakhon Ratchasima | 4 | N35 | (6) |
| *Ehrlichia* | *Haemaphysalis* | individual | 0.2 | NA | Chon Buri | 2 | N10 | (7) |
| *Ehrlichia* | *Haemaphysalis* | pool | 1.3 | NA | Nakhon Ratchasima | 4 | N35 | (6) |
| *Ehrlichia* | *Rhipicephalus* | pool | 4.9 | NA | Narathiwat | 3 | E113 | (12) |
| *Ehrlichia* | *Rhipicephalus* | pool | 0.7 | NA | Phayao | 1 | E113 | (12) |
| *Ehrlichia* | *Rhipicephalus* | individual | 21 | NA | Khon Kaen | 4 | E47 | (30) |
| *Ehrlichia* | *Rhipicephalus* | individual | 22 | NA | Kanchanaburi | 1 | N123 | (2) |
| *Ehrlichia* | *Rhipicephalus* | individual | 3.29 | NA | Bangkok | 1 | N58 | (31) |
| *Ehrlichia* | *Rhipicephalus* | individual | NA | NA | Khon Kaen, Mukdahan | 4 | N65 | (15) |
| *Ehrlichia* | *Rhipicephalus* | individual | 82 | NA | Maha Sarakham, Amnat Chareon, Nakhon Ratchasima, Bangkok | 4,1 | E68 | (17) |
| *Escherichia* | *Rhipicephalus* | individual | NA | NA | Loei | 4 | N65 | (15) |
| Eubacterium | *Haemaphysalis* | individual | 3.6 | NA | Kanchanaburi | 1 | N123 | (2) |
| Eubacterium | *Rhipicephalus* | pool | 100 | NA | Bangkok | 1 | N28 | (28) |
| Eubacterium | *Rhipicephalus* | individual | 78.6 | NA | Phra Nakhon Si Ayutthaya | 1 | N28 | (28) |
| Eubacterium | *Rhipicephalus* | pool | 100 | NA | Phatthalung | 3 | N28 | (28) |
| Eubacterium | *Rhipicephalus* | pool | 33.3 | NA | Ranong | 2 | N28 | (28) |
| *Faecalibacterium* | *Rhipicephalus* | individual | NA | NA | Roi Et | 4 | N65 | (15) |
| *Francisella* | *Rhipicephalus* | individual | 20 | NA | Surat Thani | 3 | N98 | (32) |
| *Midichloria* | *Rhipicephalus* | individual | NA | NA | Surat Thani | 3 | W11 | (33) |
| *Mycoplasma* | *Rhipicephalus* | individual | 10.6 | NA | Bangkok | 1 | E75 | (11) |
| *Pantoea* | *Rhipicephalus* | individual | NA | NA | Khon Kaen | 4 | N65 | (15) |
| *Psuedomonas* | *Rhipicephalus* | individual | NA | NA | Roi Et | 4 | N65 | (15) |
| *Rickettsia* | *Amblyomma* | individual | 26.7 | NA | Nakhon Nayok | 1 | N12 | (4) |
| *Rickettsia* | *Amblyomma* | pool | 83.6 | NA | Nakhon Ratchasima | 4 | N35 | (6) |
| *Rickettsia* | *Amblyomma* | individual | 100 | NA | Chaiyaphum | 4 | N4 | (23) |
| *Rickettsia* | *Amblyomma* | individual | 30 | NA | Nakhon Ratchasima | 4 | N71 | (34) |
| *Rickettsia* | *Amblyomma* | individual | 18.2 | NA | Nakhon Ratchasima | 4 | N73 | (3) |
| *Rickettsia* | *Dermacentor* | individual | 12.5 | NA | Phetchaburi | 2 | N12 | (4) |
| *Rickettsia* | *Dermacentor* | individual | 9.1 | NA | Tak | 1 | N12 | (4) |
| *Rickettsia* | *Dermacentor* | individual | 11.1 | NA | Kanchanaburi | 1 | N12 | (4) |
| *Rickettsia* | *Dermacentor* | individual | 33.3 | NA | Phangnga | 3 | N12 | (4) |
| *Rickettsia* | *Dermacentor* | pool | 2.7 | NA | Nakhon Ratchasima | 4 | N35 | (6) |
| *Rickettsia* | *Dermacentor* | individual | 6.1 | NA | Tak | 1 | N38 | (35) |
| *Rickettsia* | *Dermacentor* | individual | 1.7 | NA | Nakhon Ratchasima | 4 | N73 | (3) |
| *Rickettsia* | *Haemaphysalis* | pool | NA | 0.22 | Chon Buri | 2 | E19 | (1) |
| *Rickettsia* | *Haemaphysalis* | pool | 11.4 | NA | Nakhon Ratchasima | 4 | N35 | (6) |
| *Rickettsia* | *Haemaphysalis* | pool | 2 | NA | Phangnga | 3 | N42 | (36) |
| *Rickettsia* | *Haemaphysalis* | individual | 9.3 | NA | Nakhon Ratchasima | 4 | N61 | (37) |
| *Rickettsia* | *Haemaphysalis* | individual | 20 | NA | Nakhon Ratchasima | 4 | N71 | (34) |
| *Rickettsia* | *Haemaphysalis* | individual | 11.43 | NA | Chachoengsao | 1 | N71 | (34) |
| *Rickettsia* | *Haemaphysalis* | individual | 7.6 | NA | Nakhon Ratchasima | 4 | N73 | (3) |
| *Rickettsia* | *Haemaphysalis* | pool | 50 | NA | Loei | 4 | N91 | (38) |
| *Rickettsia* | *Ixodes* | pool | 16.7 | NA | Nakhon Ratchasima | 4 | N35 | (6) |
| *Rickettsia* | *Ixodes* | individual | 2.4 | NA | Nakhon Ratchasima | 4 | N84 | (39) |
| *Rickettsia* | *Rhipicephalus* | pool | 0 | NA | Chachoengsao | 1 | N16 | (20) |
| *Wolbachia* | *Haemaphysalis* | pool | 0.2 | NA | Chon Buri | 2 | N10 | (7) |
| *Wolbachia* | *Rhipicephalus* | pool | 0.5 | NA | Narathiwat | 3 | E113 | (12) |
| *Wolbachia* | *Rhipicephalus* | individual | 0.66 | NA | Bangkok | 1 | N58 | (31) |

References for S1 Table.

1. Wechtaisong W, Sri-in C, Thongmeesee K, Riana E, Bui TTH, Bartholomay LC, et al. Diversity of questing ticks and prevalence of tick-associated pathogens in Khao Kheow-Khao Chomphu Wildlife Sanctuary, Chon Buri, Thailand. Current Research in Parasitology & Vector-Borne Diseases. 2024;6:100220.

2. Parola P, Cornet J-P, Sanogo Yibayiri O, Miller RS, Thien Huynh V, Gonzalez J-P, et al. Detection of Ehrlichia spp., Anaplasma spp., Rickettsia spp., and Other Eubacteria in Ticks from the Thai-Myanmar Border and Vietnam. Journal of Clinical Microbiology. 2003;41(4):1600-8.

3. Takhampunya R, Sakolvaree J, Chanarat N, Youngdech N, Phonjatturas K, Promsathaporn S, et al. The Bacterial Community in Questing Ticks From Khao Yai National Park in Thailand. Front Vet Sci. 2021;8:764763.

4. Nooroong P, Trinachartvanit W, Baimai V, Ahantarig A. Phylogenetic studies of bacteria (Rickettsia, Coxiella, and Anaplasma) in Amblyomma and Dermacentor ticks in Thailand and their co-infection. Ticks Tick Borne Dis. 2018;9(4):963-71.

5. Sri-In C, Thongmeesee K, Wechtaisong W, Yurayart N, Rittisornthanoo G, Akarapas C, et al. Tick diversity and molecular detection of Anaplasma, Babesia, and Theileria from Khao Kheow open zoo, Chonburi Province, Thailand. Front Vet Sci. 2024;11:1430892.

6. Chaorattanakawee S, Tachavarong W, Hananantachai H, Bunsermyos W, Chanarat N, Promsathaporn S, et al. Seasonal pattern of questing ticks and prevalence of pathogenic Rickettsia and Anaplasmataceae in Khao Yai national park, Thailand. Travel Med Infect Dis. 2024;58:102696.

7. Wattanamethanont J, Kaewthamasorn M, Tiawsirisup S. Natural infection of questing ixodid ticks with protozoa and bacteria in Chonburi Province, Thailand. Ticks and Tick-borne Diseases. 2018;9(3):749-58.

8. Hirunkanokpun S, Ahantarig A, Baimai V, Pramual P, Rakthong P, Trinachartvanit W. Spotted fever group Rickettsia, Anaplasma and Coxiella-like endosymbiont in Haemaphysalis ticks from mammals in Thailand. Veterinary Research Communications. 2022;46(4):1209-19.

9. Panthawong A, Grieco JP, Ngoen-Klan R, Chao CC, Chareonviriyaphap T. Detection of Anaplasma spp. and Bartonella spp. from wild-caught rodents and their ectoparasites in Nakhon Ratchasima Province, Thailand. J Vector Ecol. 2020;45(2):241-53.

10. Aung A, Narapakdeesakul D, Arnuphapprasert A, Nugraheni YR, Wattanachant C, Kaewlamun W, et al. Multi-locus sequence analysis of Anaplasma bovis in goats and ticks from Thailand, with the initial identification of an uncultured Anaplasma species closely related to Anaplasma phagocytophilum-like 1. Comparative Immunology, Microbiology and Infectious Diseases. 2024;109:102181.

11. Do T, Phoosangwalthong P, Kamyingkird K, Kengradomkij C, Chimnoi W, Inpankaew T. Molecular Detection of Tick-Borne Pathogens in Stray Dogs and Rhipicephalus sanguineus sensu lato Ticks from Bangkok, Thailand. Pathogens. 2021;10(5):561.

12. Wongkamchai S, Perklord A, Foongladda S, Sarasombath P, Taweechue K, Loymek S, et al. Molecular identification of tick-borne pathogens and seasonal pattern of tick load on cattle in Thailand. New Biotechnology. 2016;33:S173.

13. Takada N, Fujita H, Kawabata H, Ando S, Sakata A, Takano A, et al. Spotted fever group Rickettsia sp. closely related to Rickettsia japonica, Thailand. Emerg Infect Dis. 2009;15(4):610-1.

14. Thinnabut K, Rodpai R, Sanpool O, Maleewong W, Tangkawanit U. Genetic diversity of tick (Acari: Ixodidae) populations and molecular detection of Anaplasma and Ehrlichia infesting beef cattle from upper-northeastern Thailand. Infect Genet Evol. 2023;107:105394.

15. Thanchomnang T, Rodpai R, Thinnabut K, Boonroumkaew P, Sadaow L, Tangkawanit U, et al. Characterization of the bacterial microbiota of cattle ticks in northeastern Thailand through 16S rRNA amplicon sequencing. Infect Genet Evol. 2023;115:105511.

16. Aung A, Kaewlamun W, Narapakdeesakul D, Poofery J, Kaewthamasorn M. Molecular detection and characterization of tick-borne parasites in goats and ticks from Thailand. Ticks and Tick-borne Diseases. 2022;13(3):101938.

17. Juasook A, Siriporn B, Nopphakhun N, Phetpoang P, Khamyang S. Molecular detection of tick-borne pathogens in infected dogs associated with Rhipicephalus sanguineus tick infestation in Thailand. Vet World. 2021;14(6):1631-7.

18. Saengsawang P, Kaewmongkol G, Phoosangwalthong P, Chimnoi W, Inpankaew T. Detection of zoonotic Bartonella species in ticks and fleas parasitizing free-ranging cats and dogs residing in temples of Bangkok, Thailand. Vet Parasitol Reg Stud Reports. 2021;25:100612.

19. Billeter SA, Sangmaneedet S, Kosakewich RC, Kosoy MY. Bartonella species in dogs and their ectoparasites from Khon Kaen Province, Thailand. Southeast Asian J Trop Med Public Health. 2012;43(5):1186-92.

20. Saba Villarroel PM, Chaiphongpachara T, Nurtop E, Laojun S, Pangpoo-Nga T, Songhong T, et al. Seroprevalence study in humans and molecular detection in Rhipicephalus sanguineus ticks of severe fever with thrombocytopenia syndrome virus in Thailand. Sci Rep. 2024;14(1):13397.

21. Takhampunya R, Thaloengsok S, Tippayachai B, Promsathaporn S, Leepitakrat S, Gross K, et al. Retrospective Survey of Borrelia spp. From Rodents and Ticks in Thailand. J Med Entomol. 2021;58(3):1331-44.

22. Takhampunya R, Longkunan A, Somchaimongkol S, Youngdech N, Chanarat N, Sakolvaree J, et al. Borrelia miyamotoi a neglected tick-borne relapsing fever spirochete in Thailand. PLOS Neglected Tropical Diseases. 2023;17(2):e0011159.

23. Usananan P, Kaenkan W, Sudsangiem R, Baimai V, Trinachartvanit W, Ahantarig A. Phylogenetic Studies of Coxiella-Like Bacteria and Spotted Fever Group Rickettsiae in Ticks Collected From Vegetation in Chaiyaphum Province, Thailand. Frontiers in Veterinary Science. 2022;Volume 9 - 2022.

24. Nooma W, Kaenkan W, Trinachartvanit W, Baimai V, Ahantarig A. Molecular prevalence of Coxiella like endosymbionts and the first record of Coxiella burnetii in hard ticks from Southern Thailand. Scientific Reports. 2025;15(1):10129.

25. Trinachartvanit W, Maneewong S, Kaenkan W, Usananan P, Baimai V, Ahantarig A. Coxiella-like bacteria in fowl ticks from Thailand. Parasites & Vectors. 2018;11(1):670.

26. Arthan W, Sumrandee C, Hirunkanokpun S, Kitthawee S, Baimai V, Trinachartvanit W, et al. Detection of Coxiella-like endosymbiont in Haemaphysalis tick in Thailand. Ticks Tick Borne Dis. 2015;6(1):63-8.

27. Ahantarig A, Trinachartvanit W, Wutha W, Kaenkan W, Chelong I-A, Bahakheeree M, et al. Co-infection with coxiella-like bacteria and babesia in goat ticks from southern Thailand. The Southeast Asian journal of tropical medicine and public health. 2019;50:643-50.

28. Trinachartvanit W, Kaenkan W, Nooma W, Jeangkhwoa P, Rakthong P, Baimai V, et al. Novel phlebovirus-like-AYUT and Stenotrophomonas maltophilia bacterial co-infection in a Rhipicephalus sanguineus s.l. tick. Veterinary Research Communications. 2022;46(1):277-82.

29. Muramatsu Y, Usaki N, Thongchai C, Kramomtong I, Kriengsak P, Tamura Y. Seroepidemiologic survey in Thailand of Coxiella burnetii infection in cattle and chickens and presence in ticks attached to dairy cattle. Southeast Asian J Trop Med Public Health. 2014;45(5):1167-72.

30. Eamudomkarn C, Pitaksakulrat O, Boueroy P, Thanasuwan S, Watwiengkam N, Artchayasawat A, et al. Prevalence of Ehrlichia-, Babesia-, and Hepatozoon-infected brown dog ticks in Khon Kaen Province, Northeast Thailand. Vet World. 2022;15(7):1699-705.

31. Foongladda S, Inthawong D, Kositanont U, Gaywee J. Rickettsia, Ehrlichia, Anaplasma, and Bartonella in ticks and fleas from dogs and cats in Bangkok. Vector Borne Zoonotic Dis. 2011;11(10):1335-41.

32. Rakthong P, Ruang-Areerate T, Baimai V, Trinachartvanit W, Ahantarig A. Francisella-like endosymbiont in a tick collected from a chicken in southern Thailand. Southeast Asian J Trop Med Public Health. 2016;47(2):245-9.

33. Trinachartvanit W, Rakthong P, Baimai V, Ahantarig A. Candidatus midichloria sp in a rhipicephalus sanguineus sL nymphal tick collected from a cat in thailand. Southeast Asian Journal of Tropical Medicine and Public Health. 2018;49(2):251-5.

34. Hirunkanokpun S, Kittayapong P, Cornet JP, Gonzalez JP. Molecular evidence for novel tick-associated spotted fever group rickettsiae from Thailand. J Med Entomol. 2003;40(2):230-7.

35. Chaloemthanetphong A, Ahantarig A, Apanaskevich DA, Hirunkanokpun S, Baimai V, Trinachartvanit W. A novel Rickettsia, Candidatus Rickettsia takensis, and the first record of Candidatus Rickettsia laoensis in Dermacentor from Northwestern Thailand. Scientific Reports. 2023;13(1):10044.

36. Chaorattanakawee S, Korkusol A, Tippayachai B, Promsathaporn S, Poole-Smith BK, Takhampunya R. Amplicon-Based Next Generation Sequencing for Rapid Identification of Rickettsia and Ectoparasite Species from Entomological Surveillance in Thailand. Pathogens. 2021;10(2):215.

37. Ahantarig A, Malaisri P, Hirunkanokpun S, Sumrandee C, Trinachartvanit W, Baimai V. Detection of Rickettsia and a novel Haemaphysalis shimoga symbiont bacterium in ticks in Thailand. Curr Microbiol. 2011;62(5):1496-502.

38. Hirunkanokpun S, Ahantarig A, Baimai B, Pramual P, Trinachartvanit W. A new record of Rickettsia japonica in ticks infesting a Burmese ferret-badger in Thailand. Trop Biomed. 2022;39(1):55-9.

39. Kollars TM, Jr., Tippayachai B, Bodhidatta D. Short report: Thai tick typhus, Rickettsia honei, and a unique rickettsia detected in Ixodes granulatus (Ixodidae: Acari) from Thailand. Am J Trop Med Hyg. 2001;65(5):535-7.
